# Metabolomics Analysis Reveals the Effects of Compound Fuzhuan Brick Tea (CFBT) on Regulating Dyslipidemia and Metabolic Disorders in Mice Induced by High-Fat Diet

**DOI:** 10.3390/nu14061128

**Published:** 2022-03-08

**Authors:** Xiaolu Zhou, Binggang Ge, Xuwen Zhang, Kunbo Wang, Caibi Zhou, Donghe Fu

**Affiliations:** 1 Key Laboratory of Tea Science of Ministry of Education, Hunan Agricultural University, Changsha 410128, China; arainbowl@163.com (X.Z.); gang@stu.hunau.edu.cn (B.G.); zhangxuwen@stu.hunau.edu.cn (X.Z.); wangkunbo@hunau.edu.cn (K.W.); 2 College of Biological Science and Agriculture, Qiannan Normal University for Nationalities, Duyun 558000, China; 3 School of Crop Production Technology, Institute of Agriculture Technology, Suranaree University of Technology, Nakhorn Ratchasima 30000, Thailand

**Keywords:** compound Fuzhuan brick tea, hyperlipidemia, lipid lowering, metabolic disorders

## Abstract

Background: It is well known that obesity induced by high-fat diet (HFD) poses a serious threat to people’s health. Fuzhuan brick tea, one of the most popular beverages, is reported to possess a significant effect on regulating lipid metabolism, attributed to its many bioactive ingredients. However, the efficacy and mechanism of compound Fuzhuan brick tea (CFBT) made from Fuzhuan brick tea and other six Chinese herbal medicines are still not well defined. Methods: Sixty mice were divided into six groups: normal control group (CK), high-fat model group (NK), positive control group with anti-hyperlipidemic drug (YK), CFBT at low-(FL), medium-(FM) and high-(FH) dosage. Intervening for 30 days, conventional indexes analysis combined with metabolomics were performed to evaluate the changes in biochemical indexes and liver metabolic profiles in mice submitted to HFD. Results: CFBT treatment was able to ameliorate obesity, serum biochemical parameters, antioxidant activity and hepatic steatosis. In addition, significant alterations in the liver tissue metabolic profiles were observed, with most of these associated with inflammation, glucose and lipid metabolism. Conclusions: This study provides evidence that consumption of CFBT is capable of preventing dyslipidemia, reducing weight gain, restoring liver injury, as well as improving metabolic disorders.

## 1. Introduction

The continuous ingestion of a high-energy and high-fat diet (HFD) may cause harmful activation of the immune system and induce lipid metabolism disorder, which may lead to chronic metabolic diseases such as diabetes, hypertension, cardiovascular disease and obesity [1,2]. The prevalence of obesity has doubled in more than 70 countries and has continued to increase in most other countries over the past several decades [3], becoming a significant global public health problem [4,5,6]. The disorder of fat metabolism in obese individuals is mainly manifested by the increase in plasma free fatty acids, cholesterol, triglycerides, total fat and other lipid profiles. When suffering from obesity, the reduction in mobilization and utilization of free fatty acids is accompanied by the elevation of free fatty acids and lipid volume in the blood, and patients with carbohydrate-induced hypertriglyceridemia are prone to obesity. Obesity is closely associated with hyperlipidemia. The level of triglyceride in the blood of obese patients is increased. Meanwhile, the fasting and postprandial insulin of obese patients will be increased, which may be responsible for the increase in fat synthesis and the decrease in fat decomposition. Therefore, obese patients are usually accompanied by high blood lipids. There are two types of hyperlipidemias: primary hyperlipidemia is related to genetic factors and abnormalities of enzymes, receptors or apolipoproteins involved in lipoprotein metabolism and transport due to genetic defects; secondary hyperlipidemia is mostly caused by metabolic disorders (diabetes, obesity, hypertension, liver and kidney diseases, etc.). With the continuous innovation and development of plant natural products and medical research, natural drugs and high-efficiency health care products made of plant functional ingredients have strong biological activity [7,8], which can achieve high efficiency in treating diseases, but also have side effects on the human body [9].

Many conventional treatments for the prevention of obesity have proved to be ineffective; considerable attention has been focused on tea consumption because it is rich in several bioactive substances, such as tea polyphenols, catechins, caffeine, tea polysaccharides and other potential biologically active compounds [10,11], which are able to effectively reduce the content of triglycerides (TG) and cholesterol, promote the excretion of TG from feces [12], and improve oxidase activity [13]. Numerous reports have shown that the beneficial components of tea can promote a reduction in body weight by regulating the expression of multiple genes in adipose tissue of obese mice induced by HFD [14,15,16,17], decrease glucose concentration and leptin level [18], and modify the gut microbiota [19]. Li Qiuhua et al. found that stir-fried tea (HT) could decrease the levels of liver and serum TG and that the degree of hepatic steatosis and adipocyte hypertrophy was accompanied by weight loss. Meanwhile, HT treatment was seen to have a potential protective effect on obese mice induced by a HFD via activating AMPK/ACC/CPT1 pathway and plays a significant protective role to a certain extent [20]. Similarly, another study reported that green tea extract (GTE) intake could decrease body weight, prevent the accumulation of liver fat and ameliorate hyperglycemia and insulin resistance in HFD-fed mice. Moreover, these anti-obesity mechanisms suggested that GTE supplementation may improve fatty liver and weight gain in hyperlipidemic mice by activating sirtuin 1 and amp-activated protein kinase pathways [21]. In addition, the daily consumption of tea has been reported to effectively inhibit intestinal lipid absorption, reduce liver fat tissue content and pancreatic lipase activity and improve free fatty acid induced liver cell insulin resistance related metabolic abnormalities, thereby regulating blood lipid levels and preventing obesity or type 2 diabetes caused by HFD [22,23], as well as reducing a variety of diseases and health risks [24,25,26,27,28].

Metabonomics provide a new perspective for drug discovery [29], drug toxicity evaluation [30], disease diagnosis [31], pathogenetic mechanisms and several related topics, as its aim is the quantitative and qualitative analysis of the dynamic metabolic response of biological systems to biological stimulation and genetic modification [32]. Moreover, metabonomic is highly effective for identifying metabolic changes caused by environmental chemical exposure and screening molecular biomarkers. Fuzhuan brick tea, a popular post-fermented tea consumed as a daily beverage by a large number of people from Hunan Province in China, has been widely applied in food, medical and cosmetic industries due to its unique quality, flavor [33] and complex chemical substances [34,35] formed by special processing technology [36], as well as its remarkable health care effects. In our previous paper, a compound teabag with Fuzhuan brick tea as the main raw material was developed, which had a unique flavor and high safety [37]. In recent years, some studies have revealed the efficiency of Fuzhuan brick tea [38,39], but there are few reports on CFBT. In this study, metabonomics and regular serum biochemical detection methods were used to analyze the biochemical abnormalities of mice induced by HFD and evaluate the therapeutic effect of CFBT.

## 2. Materials and Methods

### 2.1. CFBT Preparation and Extraction

All the plant materials were obtained from Hunan Changsha Dekang Biotechnology Co., Ltd. (Changsha, China). CFBT is made of Fuzhuan brick tea (*Camellia sinensis*), hawthorn (*Crataegus pinnatifida* Bge.), dandelion root (*Taraxacum mongolicum* Hand.-Mazz.), mint leaves (*Mentha canadensis* L.), fennel (*Foeniculum vulgare* Mill.), Orange peel (*Citrus reticulata* Bla.) and chamomile (*Matricaria chamomilla* L.), with a weight ratio of 5:5:5:6:3:5:4. An appropriate amount of CFBT samples were ground and extracted in boiling water at a ratio of 1:10 for 30 min, and then filtered with two layers of industrial gauze after the tea infusion was slightly cold. The tea dregs were extracted again for 20 min in water (1:8, *w*/*v*), and the tea infusion extracted twice was mixed and filtered by vacuum. The filtrate was concentrated to a certain concentration with a rotary evaporator. After being frozen at −80 °C for 12 h, the samples were freeze-dried for 28 h. Finally, the dried powder was collected and stored at −80 °C before use.

### 2.2. Animal Experimental Design

Sixty 5-week-old SPF male Kunming mice (license number SCXK (Xiang) 2016-0002) weighted 32 ± 2 g were purchased from Hunan Slack Jingda Experimental Animal Co., Ltd. (Changsha, China). The mice were kept in a room with a 12 h day and night cycle at a temperature of 20–26 °C and a relative humidity of 50–60%. After 7 days of adaptive feeding, the mice were randomly divided into 6 groups with 10 mice in each group, and they were labeled. The normal control group (CK) was fed a normal diet, while other groups were fed a high-fat diet (1.5% cholesterol, 0.5% bile salt, 5.0% egg yolk powder, 10.0% lard, and 83.0% normal diet). The CK and model control groups (NK) were given the same volume of mineral water, while the mice fed a high-fed diet were treated with Xuezhikang capsule (YK, 91.0 mg/kg), CFBT at low-(FL, 247.5 mg/kg), medium-(FM, 495.0 mg/kg) and high-dosage (FH, 990.0 mg/kg) for 4 consecutive weeks. During the experiment, the food intake and activity status of the mice were observed and recorded every day, and the mice were weighed every week.

### 2.3. Serum Collection and Biochemical Analysis

The blood samples were collected in a 2 mL EP tube after the experiment and kept at room temperature for 30 min to avoid vibration, and then were centrifuged at 3500× *g* at 4 °C for 10 min to obtain the upper serum. The serum total cholesterol (TC), triglyceride (TG), low-density lipoprotein cholesterol (LDL-C), high-density lipoprotein cholesterol (HDL-C), glucose, insulin, alanine aminotransferase (ALT) and aspartate aminotransferase (AST) were determined by using kits purchased from Nanjing Jiancheng Institute of Bioengineering (Nanjing, China).

### 2.4. Preparation of Liver Tissue Homogenate

The mice liver tissues were rinsed in normal saline to remove blood. After drying, 0.2 g was weighed and added with normal saline to grind on ice bath to prepare 10% liver tissue homogenate and centrifuged at 3500× *g* at 4 °C for 10 min, and then the supernatant was collected and subpackaged. Superoxide dismutase (SOD) and malondialdehyde (MDA) in liver homogenate were determined with kits according to the manufacturer’s instructions (provided by Nanjing Jiancheng Institute of Bioengineering, Nanjing, China).

### 2.5. Histopathology Examination

About 0.5 g of liver tissue was immersed in 10% formalin solution for 48 h, embedded in paraffin and sliced into 5μm thickness. The slices were placed in xylene for 20 min and dehydrated in 100%, 95%, 85% and 75% ethanol for 5 min successively. Then, the slices were rinsed with distilled water for 5 min and stained with hematoxylin and eosin (H&E) for 1 min, respectively. The dried slices were placed in xylene for 10 min and sealed with neutral gum. The H&E staining images were captured at 40-times magnification.

### 2.6. Samples Preparation for Metabolomics

A 50 mg fresh tissue sample was homogenized with 1000 μL ice cold methanol/water (70%, *v*/*v*), then the cold steel balls were added to the mixture and homogenized at 30 Hz for 3 min. After the mixture was vortexed for 1 min, and centrifuged at 12,000× *g* for 10 min at 4 °C. Subsequently, the collected supernatant was used for LC-MS/MS analysis.

### 2.7. HPLC-MS Analysis

LC-ESI-MS/MS system (UPLC, Shim-pack UFLC SHIMADZU CBM A system, https://www.shimadzu.com/, accessed on 17 September 2019; MS, QTRAP^®^ 6500 + System, https://sciex.com/, accessed on 17 September 2019) was used to analyze the samples. The samples were injected into a 2 μL of ACQUITY UPLC HSS T3 C18 column (1.8 µm, 2.1 mm × 100 mm, Waters); Solvent system was water (0.04% acetic acid, mobile phase A): acetonitrile (0.04% acetic acid, mobile phase B); Gradient program was shown in Table 1. Mass spectra were analyzed and obtained by using a triple quadrupole-linear ion trap mass spectrometer (QTRAP^®^ 6500 + LC-MS/MS System) equipped with an ESI Turbo Ion-Spray interface with positive and negative ion mode and controlled by Analyst 1.6.3 software (Sciex). The temperature of source was set at 500 °C. Ion spray voltage (IS) was set at 5500 V (positive) and −4500 V (negative). The ion source gas I (GSI), gas II (GSII) and curtain gas (CUR) were set at 55, 60, and 25.0 psi, respectively. A total of 10 and 100 μmol/L polypropylene glycol solutions were used for instrument tuning and mass calibration in QQQ and LIT modes, respectively.

### 2.8. Data Processing and Statistical Analysis

All data were submitted to one-way analysis of variance using the statistical software SAS 9.0. Data are presented as means ± SE. *p*-value > 0.05 means the difference is not significant, *p*-value < 0.05 is accepted as significant, and *p*-value < 0.01 is considered to be a significant difference.

All of the LC/MS data were processed by using MVDB V2.0 database (Wuhan Metware Biotechnology Co., Ltd., Wuhan, China) and public database. After the metabolites were quantitatively analyzed by triple quadrupole mass spectrometry multi reaction monitoring mode (MRM), the qualitative and quantitative analysis of mass spectrometry was performed by the Software Analyst 1.6.1, including baseline filtration, peak identification, integration, retention time correction, peak alignment and mass spectrometry fragment attribution analysis. The data was normalized and annotated according to the obtained retention time, mass-to-charge ratio and peak intensity, and further confirmed the use of standards for most substances. Firstly, the principal component analysis (PCA) of unsupervised pattern recognition was used to analyze the detected metabolites to obtain a preliminary understanding of the overall metabolite difference between the samples of each group and the degree of variability between the samples within the group. In addition, partial least squares-discriminant analysis (PLS-DA) with supervised pattern recognition was used to distinguish the overall difference of metabolites between groups to find different metabolites. Cross-partial least squares discriminant analysis (OPLS-DA) was used to remove irrelevant differences to screen different variables to find differences between samples in each group, which was extensively used to select the biomarkers by a variable importance in projection (VIP). The validity of the models against overfitting was estimated by the parameter R^2^Y values, and the predictive ability was described by Q^2^ values. Furthermore, the differential metabolites were screened by multidimensional statistics VIP value (VIP > 1), one-dimensional Statistics (*p* < 0.05) and fold change transformed by Log_2_. The metabolites with VIP > 1, *p* < 0.05, log_2_FC ≥ 2 or log_2_FC ≤ 0.5 were selected as differential metabolites.

## 3. Results

### 3.1. The Effects of CFBT on Body Weight and Liver Weight

**Figure 1:**
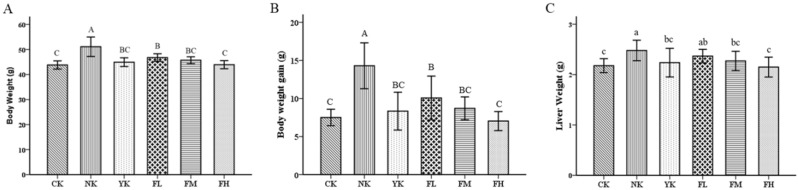
CFBT prevents the increase in body weight and liver weight. (**A**) Body weight, (**B**) body weight gain and (**C**) liver weight. CK, control group; NK, high-fat diet group; YK, high-fat diet with positive drug Xuezhikang; FL, high-fat diet with low-dosage CFBT; FM, high-fat diet with medium-dosage CFBT; FH, high-fat diet with high-dosage CFBT. All data are presented as the mean ± SD (*n* = 10/group). Different lowercase letters above the bars showed significant difference at 0.05 level among the same data series; different capital letters showed highly significant difference at 0.01 level among the same data series.

To investigate the efficiency of reducing blood lipids of CFBT on HFD-induced mice, male mice were given by HFD and treated with 247.5 mg/kg, 495.0 mg/kg and 990.0 mg/kg of CFBT for 30 consecutive days. After the experiment, the mice were weighed, and the organs were removed for weighing. A significant increase was observed in the body weight of mice fed with HFD and drank water (*p* < 0.01) (Figure 1A), indicating that the high-fat model mice were successfully constructed, which was dramatically different from the CK group and other experimental groups (*p* < 0.01) (Figure 1A). As shown in Figure 1B, the body weight gain in the NK group was 1.9-fold higher than that of CK group. Meanwhile, 30 days of treatment with low-, medium-, and high- dose CFBT or lipid-lowering drugs significantly controlled the weight gain of mice, which were significantly reduced by 29.6%, 39.2%, 50.8% and 41.7%, respectively, compared to the NK group. Similarly, there was an increase in the liver weight of mice exposed to the HFD, while the liver weight of mice in the CK group and other experimental groups was significantly lower than that of the NK group (*p* < 0.05) (Figure 1C). The results showed that CFBT and Xuezhikang capsule treatment were able to prevent the increase in body weight and liver weight in mice, and the effect of preventing weight gain was more obvious with the increase in dosage of CFBT.

### 3.2. CFBT Improves Serum Biomarkers and Oxidative Stress

**Figure 2:**
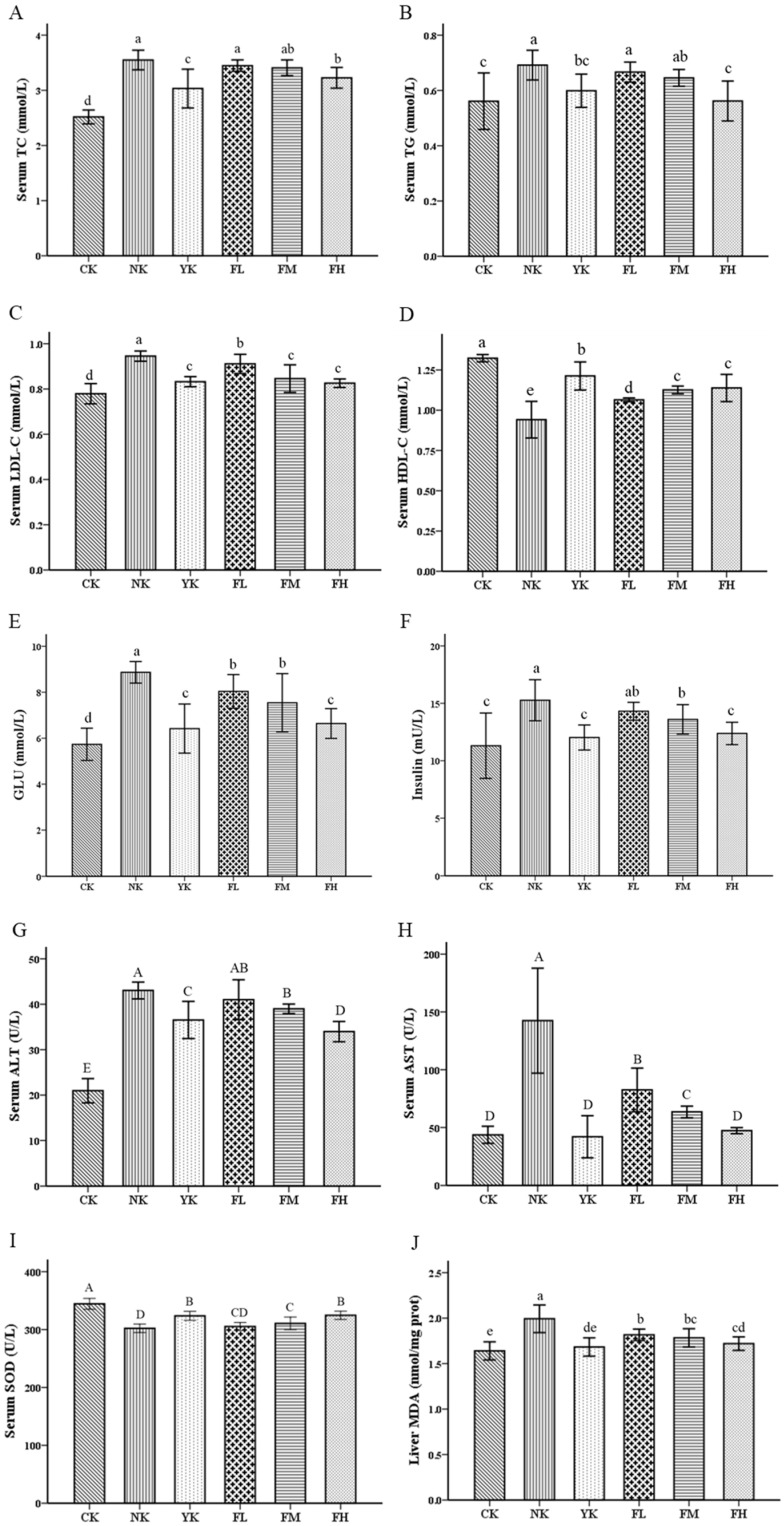
CFBT improves serum biochemical indexes. (**A**) Serum total cholesterol (TC), (**B**) serum triglyceride (TG), (**C**) serum low-density lipoprotein cholesterol (LDL-C), (**D**) serum high-density lipoprotein cholesterol (HDL-C), (**E**) serum glucose (GLU), (**F**) serum insulin, (**G**) serum alanine aminotransferase (ALT), (**H**) serum aspartate aminotransferase (AST), (**I**) liver superoxide dismutase (SOD) and (**J**) liver malondialdehyde (MDA). CK, control group; NK, high-fat diet group; YK, high-fat diet with positive drug Xuezhikang; FL, high-fat diet with low-dosage CFBT; FM, high-fat diet with medium-dosage CFBT; FH, high-fat diet with high-dosage CFBT. All data are presented as the mean ± SD (*n* = 10/group). Different lowercase letters above the bars showed significant difference at 0.05 level among the same data series; different capital letters showed highly significant difference at 0.01 level among the same data series.

To evaluate the lipid profile, the serum samples of mice were analyzed. There was a significant increase in the levels of serum TC, TG and LDL-C in the NK group (*p* < 0.05) (Figure 2A–C), while the level of HDL-C was lower compared to CK group (*p* < 0.05) (Figure 2D). On the contrary, the levels of serum TC, TG and LDL-C of mice ingested with different dosages of CFBT were generally lower than those of the NK group, and the level of serum HDL-C increased to a significant level (*p* < 0.05) (Figure 2A–D). However, there was no significant difference in serum levels between YK group and each CFBT group, especially compared with the high-dose group, indicating that CFBT and Xuezhikang capsule treatment could effectively reduce serum lipids of mice fed with HFD. Regarding glucose and insulin levels, there was a marked increase, as expected, in the NK group when compared with the CK group (*p* < 0.05), while treatment with CFBT and Xuezhikang were able to prevent the increase in serum glucose and insulin (Figure 2E,F), again suggesting a potential antihyperlipidemic and antidiabetic effect.

When mice were exposed to HFD, the levels of serum ALT and AST in the NK group were significantly increased compared with the CK group, indicating that the long-term intake of HFD caused some damage to the normal liver function of mice (*p* < 0.01). The serum ALT and AST levels were compared between the groups that ingested CFBT and the group fed with HFD and mineral water; a marked reduction in the consumption of different doses of CFBT was observed (*p* < 0.01) (Figure 2G,H). Furthermore, the higher the dosage of CFBT consumed by mice, the more similarities in the level of ALT and AST were observed to that of CK group (Figure 2G,H). The same was found in the YK group (Figure 2G,H). It was suggested that CFBT and Xuezhikang treatment was able to prevent the increase in serum AST and ALT levels and effectively inhibit the adverse effects of HFD on the liver of mice.

After ingestion of HFD, the serum SOD activity of the liver of mice was significantly lower than that of the CK group (*p* < 0.01), which suggested that the consumption of HFD could cause the decrease in serum SOD in the liver of mice (Figure 2I). When mice were fed with CFBT and Xuezhikang, SOD levels showed a significantly increased trend (*p* < 0.01). Ingestion of HFD increased the level of serum MDA, which led to an increase inlipid peroxidation and oxidative damage. It was seen that the NK group had a marked increase in MDA level compared to the CK group (*p* < 0.05), whereas CFBT and Xuezhikang treatment prevented the increase in MDA level and alleviated the reaction of liver lipid oxidation (Figure 2J).

### 3.3. CFBT Prevents Liver Injury on HFD Mice

**Figure 3:**
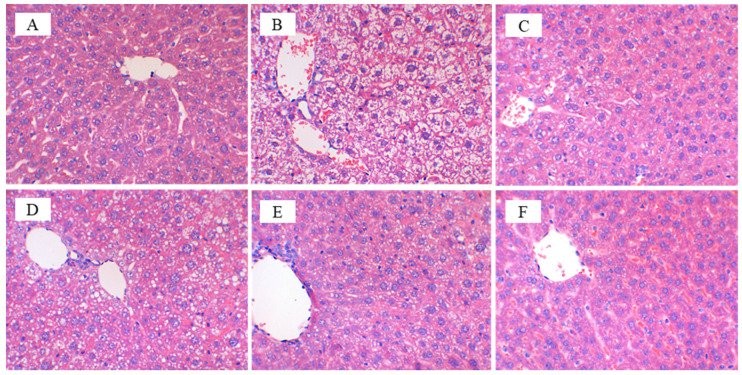
Effects of CFBT intervention on Hematoxylin-eosin staining of liver tissues. (**A**) Normal control group (CK); (**B**) High-fat model group (NK); (**C**) Positive control group (YK); (**D**) Low-dose CFBT (FL); (**E**) Medium-dose CFBT (FM); (**F**) High-dose CFBT (FH).

Morphological analyses of mice liver showed that mice that ingested only a normal diet and mineral water maintained a normal hepatocyte structure and size (Figure 3A), while in the NK group, which was exposed to HFD and mineral water, hepatocytes became larger and severe fatty lesions were present with the accumulation of lipid droplets in the cytoplasm (Figure 3B). When the mice received CFBT (Figure 3E,F), the morphology of hepatocytes was similar to that of the CK group; the lipid droplets did not assemble and the liver cells were normal, especially in the FH group. Similarly, this result was observed in the YK group fed with a positive drug Xuezhikang capsule (Figure 3C). We could conclude that the consumption of medium- and high-doses of CFBT and positive drugs had the ability to alleviate liver steatosis in mice and protect and restore the liver to a certain extent, but the effect of low-dose CFBT was not significantly obvious (Figure 3D).

### 3.4. Metabolic Effects of CFBT on HFD Mice

**Figure 4:**
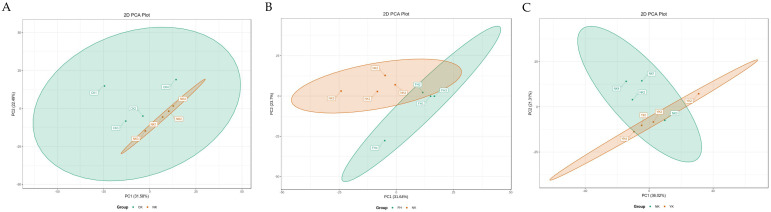
PCA plots of samples from CK group, NK group and YK group. (**A**) CK group vs. NK group; (**B**) NK group vs. FH group; (**C**) NK group vs. YK group.

Multivariate statistical analysis was used to “simplify and reduce the dimensionality” of high-dimensional and complex data on the basis of preserving the original information to the greatest extent, and a reliable mathematical model was established to summarize the characteristics of the metabolic spectrum of samples. Principal coordinate analysis (PCA) was used to evaluate the differences in the metabolites in each group. The PCA plot of samples showed the obvious separation between the CK group and NK group (Figure 4A), with 31.58% and 22.49% variation explained by the first two (PC1 and PC2) principal components, respectively, suggesting that the metabolic activities of mice induced by HFD in the NK group were systematically maladjusted compared with those in the CK group. FH and YK groups were divided into distinctive clusters with the NK group (Figure 4B,C), indicating that CFBT and Xuezhikang treatment were able to regulate metabolic disorders in the liver of mice induced by HFD.

**Figure 5:**
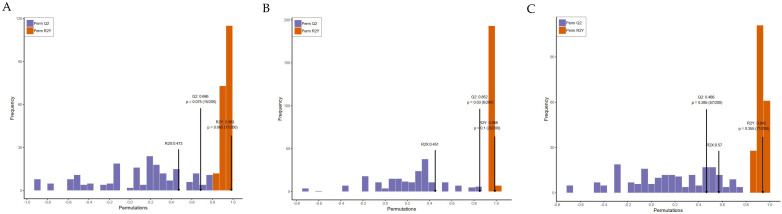
OPLS-DA model verification from CK, NK and YK groups. (**A**) CK group vs. NK group; (**B**) NK group vs. FH group; (**C**) NK group vs. YK group.

Orthogonal partial least squares discriminant analysis (OPLS-DA) of potential structures was conducted to reveal detailed and statistically significant changes in metabolites caused a by high-fat diet and related to treatment. The OPLS-DA model further showed that the R2 and Q2 parameters of each group were relatively high with good predictive ability and reliability (Figure 5), which was able to manifest the change trend in metabolites among groups. Subsequently, variable importance projection (VIP) of the obtained multivariate analysis OPLS-DA model was used for preliminary analysis and screening of differential metabolites. Simultaneously, the differential metabolites could be further screened by combining the *p*-value or fold change in univariate analysis. A total of 602 metabolites were identified in the liver tissue of mice by using the widely targeted metabolomics technique (Table 2).

**Figure 6:**
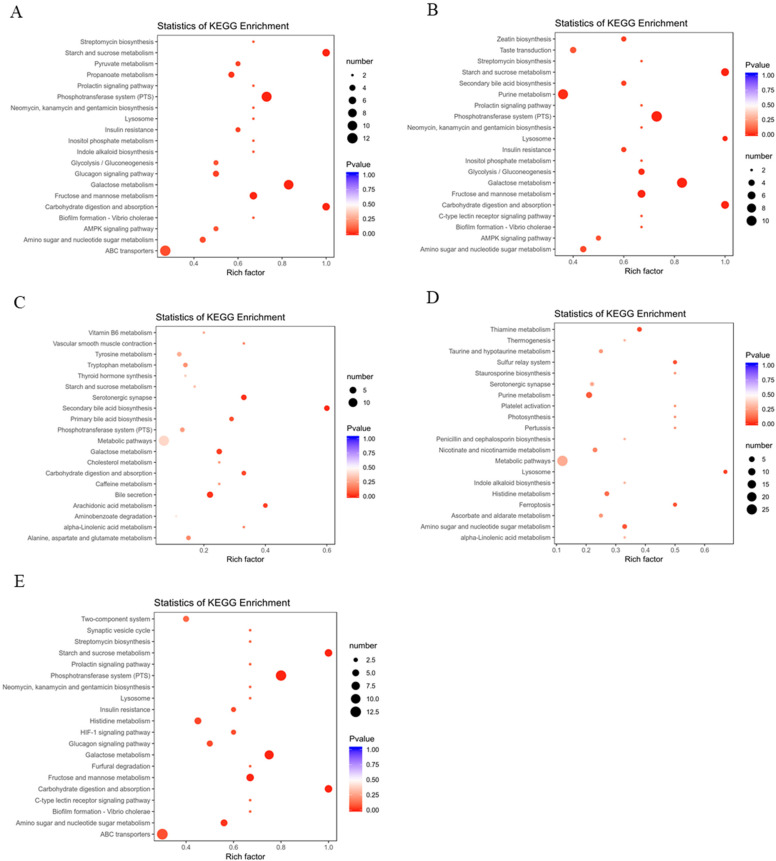
Changes in metabolites and related pathways in the liver of mice caused by HFD according to the KEGG pathway database. (**A**) CK group vs. NK group; (**B**) NK group vs. FH group; (**C**) NK group vs. YK group; (**D**) CK group vs. FH group; (**E**) CK group vs. YK group.

Compared with the CK group, the most important metabolites identified in the NK group to be up-regulated were taurodeoxycholic acid sodium salt and taurochenodesoxycholic acid, D-sorbitol, D-fructose, D-galactose, D-glucose, D-mannose, D-trehalose, D-glucose 6-phosphate, D-sedoheptuiose 7-phosphate, D-fructose 6-phosphate-disodium salt, N-Acetylglucosamine 1-phosphate, lysopg 18:1, lysope 18:1 and lysopc 20:2. The significantly down-regulated metabolites observed in the NK group were octadecatrienoic acid, eicosapentaenoic acid, eicosatetraenoic acid, Docosahexaenoic acid, 12-Hydroxyeicosane tetraenoic acid, and Punicic acid, etc., which were mainly enriched in the pathways of starch and sucrose metabolism, phosphotransferase system (PTS), pyruvate metabolism, galactose metabolism, carbohydrate digestion and absorption, and fructose and mannose metabolism (Figure 6A). In NK vs. FH group, we found that tauroursodeoxycholic acid, taurochenodesoxycholic acid, glycoursodeoxycholic acid, D-sorbitol, D-fructose, D-galactose, D-glucose, D-mannose, D-sucrose, and D-trehalose, etc. were significantly down-regulated and most chemical compounds in the the FH group: eicosapentaenoic acid, eicosatetraenoic acid, docosahexaenoic acid, and 12-hydroxyeicosane tetraenoic acid, were significantly up-regulated compared to the NK group, which were annotated in the pathways of starch and sucrose metabolism, PTS, galactose metabolism, fructose and mannose metabolism, carbohydrate digestion and absorption and AMPK signaling pathway (Figure 6B). Moreover, it showed that the positive drug Xuezhikang capsule was able to reduce hyodeoxycholic acid, glycocholic acid, deoxycholic acid, chenodeoxycholic acid, taurodeoxycholic acid sodium salt, which were enriched in primary and secondary bile acid biosynthesis, galactose metabolism, carbohydrate digestion and absorption, and bile secretion; interestingly, the increased content of D-sucrose, dulcitol, maltotriose and the decreased content of thromboxane B2, prostaglandin D2, eicosapentaenoic acid, eicosatetraenoic acid, eicosatrienoic acid, docosahexaenoic acid in pathways of glucose metabolism and arachidonic acid metabolism compared to the NK group (Figure 6C). In CK vs. FH group, the FH group showed the higher D-fructose 6-phosphate-disodium salt, D-fructose-1,6-biphos-phate-trisodium salt, 3′-sialyllactose, hydroxyphenyllactic acid, D-mannose 6-phosphate, D-fructose 6-phosphate, N-acetylglucosamine 1-phosphate, 3′-sialyllactose, galactaric acid, D-mannose 6-phosphate, lactobionic acid, maltotriose, N-Acetyl-D-glucosamine, raffinose, D(-)-threose, docosahexaenoic acid, Cis-11,14-eicosadienoic acid (C20:2) and the lower taurodeoxycholic, D-sucrose, formononetin, lactose, eicosapentaenoic acid, eicosatetraenoic acid, stearidonic acid, 2-methylbutyroylcarnitine in the pathways of purine metabolism, amino sugar and nucleotide sugar metabolism, and ascorbate and aldarate metabolism compared to the CK group (Figure 6D). In CK vs. YK group, the mice in YK group had the lower hyodeoxycholic acid, glycocholic acid, deoxycholic acid, chenodeoxycholic acid, chenodeoxycholic acid, L-fucose, L-rhamnose, formononetin, thromboxane B2, prostaglandin D2, eicosapentaenoic acid, eicosatetraenoic acid, eicosatrienoic acid, 3-methylglutaric acid, tetradecanedioic acid and the higher D-sorbitol, D-fructose, D-galactose, D-glucose, lactobionic acid, etc., which were annotated into the metabolic pathways of phosphotransferase system, starch and sucrose metabolism, galactose metabolism, fructose and mannose metabolism, carbohydrate digestion and absorption, amino sugar and nucleotide sugar metabolism and ABC transporters (Figure 6E).

## 4. Discussion

Obesity is characterized by the increased storage of triglycerides in expanded adipose tissue and is the result of an imbalance between energy intake and energy consumption. Generally, obesity is associated with many risk factors, such as hyperlipidemia, hypertension, cardiovascular disease, type-2 diabetes mellitus and insulin resistance [40,41,42]. CFBT, a folk Chinese medicine formula, has been widely applied by local people to lose weight, but its remedial mechanisms are still not well defined. The clinical trial of CFBT is the research plan we will pursue in the future. However, CFBT is just a health care medicine, which may involve a long therapeutic cycle, and the curative effect in clinic may not be as effective as that in animal experiments. In addition, another limitation of this study, in respect to clinical application, is the need to involve large-scale volunteers who we could not find in a short time. Therefore, we investigated the anti-obesity and hypolipidemic effects of CFBT on hyperlipidemic mice to determine whether CFBT could reverse dyslipidemia and metabolic abnormalities induced by HFD. Our results clearly illustrated that CFBT prevented body weight gain in mice and it is believed that it would also reduce the accumulation of fat in the body. In particular, a high dose of CFBT could significantly suppress the body weight in obese mice after a month of treatment. A study on the anti-obesity effect of Fuzhuan brick tea also showed that ingestion of Fuzhuan brick tea could reduce HFD-induced obesity significantly [43]; it was similar to this study. Clinically, four plasma lipid indexes are detected to diagnose hyperlipidemia, which is usually accompanied by an increase in the level of TC, TG, LDL and a decrease in HDL level. Many studies on the relationship between serum lipid and lipoprotein levels, atherosclerosis and coronary heart disease have been reported [44,45]. Our data demonstrated that CFBT or Xuezhikang treatment was attributed to effectively improve the balance of blood lipid, reduce the content of TC, TG and LDL in serum, and increase the content of HDL when compared with their counterparts. Similar results were found in Pu’er tea [46], a kind of dark tea, and active components of tea [47]. In terms of glucose concentration and insulin level, the mice that were submitted to an HFD presented a significant increase compared to the mice treated with a normal diet; whereas, the levels of blood glucose and insulin were decreased after all CFBT administration, indicating that CFBT treatment may maintain blood lipid homeostasis responsible for glucose and lipid metabolism via an antihyperlipidemic action.

In addition, the effects of CFBT treatment on liver physiological and biochemical indexes of mice fed with HFD were also investigated. Previous studies have shown that Fuzhuan brick tea can prevent liver steatosis and liver injury induced by HFD [48]. In our study, the liver weight of the NK group was significantly higher than that of the CK group, which was due to the hepatomegaly caused by HFD. After intragastric administration of different doses of CFBT or Xuezhikang, the liver weight of mice decreased, and the symptoms of liver enlargement and pathological changes were alleviated. From the macroscopic and microscopic observation, the liver of the NK group showed severe fatty lesions, while the liver lesions of mice were improved after different doses of CFBT treatment. It is well known that the level of ALT and AST is positively correlated with the degree of liver abnormality [49,50]. In the clinic, the value of AST/ALT is commonly used to reflect the damage of liver cells. Our results suggested that the serum levels of AST, ALT and the ratio of AST/ALT of HFD-induced mice were significantly higher than that of healthy mice, which indicated that ingestion of HFD could affect the liver function of normal mice and cause serious damage to liver cells. CFBT-treated mice displayed significantly lower serum levels of ALT and AST, indicating that CFBT could protect and restore liver cells. We also found that consumption of HFD could reduce SOD activity, exacerbate liver lipid oxidation and increase MDA content, while CFBT or Xuezhikang treatment was able to increase the activity of SOD and decrease the level of MDA. A similar study reported that the extract of Fuzhuan brick tea contributed to antioxidant activity and may also directly act as instant tea products [51]. Therefore, CFBT could be considered as an effective antioxidant to benefit health.

The metabolome in mice induced by HFD were further evaluated and it was found that the metabolism of phospholipids, fatty acids, bile acids, and glycolipids in hyperlipidemia mice was significantly disturbed. Lysophospholipid is a compound that catalyzes the fatty acid hydrolysis of phospholipid molecules by phospholipase A, which can be used as a clinical diagnostic indicator to reveal pathophysiological changes. In our study, LysoPG (18:1), LysoPE (18:1) and LysoPA (18:0) in liver tissue of hyperlipidemic mice submitted to HFD in the NK group were significantly increased compared to the CK group. Previous studies related to metabolomics have been demonstrated that significantly increased plasma. LysoPC (16:0), lysoPC (18:0), lysoPC (18:1) and lysoPC (22:5) were identified in the rat model of atherosclerosis induced by HFD [52,53], which were consistent with the results of our study. Some altered Lysophospholipids in mice that received HFD were significantly correlated with the pathogenesis of hyperlipidemia and could be recognized as potential biomarkers, while there was a complex interaction between blood lipid levels and Lysophospholipids [54]. In combination with conventional biochemical indexes of blood lipids, lysophospholipids such as lysoPG (18:1), lysoPE (18:1) and lysoPA (18:0) showed a significant positive correlation with body weight gain, liver weight and serum level of TC, TG, LDL, AST, ALT and MDA, which were accompanied by negative correlations to the serum level of HDL and SOD.

Bile acids have recently been reported to play an important role in regulating lipid metabolism and energy metabolism of the host as a signal molecule [55], contributing to lipid digestion and absorption, affecting the secretion of bile and regulating the level of cholesterol [56]. Evidently, the content of total bile acid in serum of hyperlipidemia population was positively correlated with the levels of TC and TG [57], which was consistent with the increased serum TC and TG levels in mice exposed to HFD were accompanied by the significantly increased hepatic bile acid metabolites, taurodeoxycholic acid sodium salt (TDCAS) and taurochenoxycholic acid (TCDCA), in our findings. CFBT treatment was able to decrease tauroursodeoxycholic acid (TUDCA), TCDCA and glycooursodeoxycholic acid (GUDCA), while Xuezhikang reduced hyodeoxycholic acid (HDCA), glycocholic acid (GCA), deoxycholic acid (DCA) and chenodeoxycholic acid (CDCA). Consequently, CFBT and Xuezhikang accelerated the absorption and metabolism of bile acids to lipids.

The phosphotransferase system (PTS) is involved in the transport of many sugars to bacteria, such as glucose, mannose, fructose and cellobiose [58]. Our results suggested that most of the increased carbohydrate metabolites (e.g., D-fructose, D-galactose, D-glucose, D-mannose, D- glucose 6-phosphate, L-erythrulose, etc.) in the liver of mice that consumed HFD were in the metabolic pathways of galactose, starch, sucrose, fructose and mannose, while CFBT and Xuezhikang treatment depressed carbohydrate digestion and absorption. Fatty acids were capable of inhibiting the oxidation of HDL-C and enhance the anti-atherosclerotic function of HDL-C [59]. Docosahexaenoic acid (DHA) and eicosapentaenoic acid (EPA) were able to act together with eicosatetraenoic acid (12-HETE) to produce anti-inflammatory molecules such as lipoxin. The balance between these antagonistic compounds determined the development process of chronic inflammatory diseases, including hyperlipidemia, diabetes and atherosclerosis [60]. The significantly decreased levels of EPA, DHA and 12-HETE in the NK group, as was observed in this study, broke the dynamic balance between compounds, resulting in hyperlipidemia, atherosclerosis, as well as cardiovascular and cerebrovascular diseases. Consumption of CFBT increased the levels of these metabolites and inhibited the occurrence of related diseases such as hyperlipidemia; however, Xuezhikang treatment was not responsible for the similar effects on these metabolites as CFBT treatment.

## 5. Conclusions

In summary, this study revealed the effects of CFBT exposure on serum biochemical indexes and liver metabolism in mice induced by HFD. Significant biochemical indexes and histological changes related to dyslipidemia and oxidative stress were observed in the conventional serum of hyperlipidemic mice exposed to CFBT. Significant alterations in the liver metabolic profiles were observed to be attributed to CFBT treatment, most of which were related to liver inflammation and lipid metabolism. These results showed that CFBT consumption had the ability to reduce lipid digestion, restore dyslipidemia and liver injury, as well as regulate the metabolic level caused by a high-fat diet. Therefore, our findings highlight the latent health risk of CFBT exposure to hyperlipidemia and provide insights for further investigations into its metabolic mechanism.

## Figures and Tables

**Table 1:** Elution gradient.

Time (min)	Flow Rate (mL/min)	Temperature (°C)	Mobile Phase A (V)	Mobile Phase B (V)
0.0	0.4	40	95	5
11.0	0.4	40	5	95
12.0	0.4	40	5	95
12.1	0.4	40	95	5
14.0	0.4	40	95	5

**Table 2:** Significant changes in potential metabolites were identified by LC—MS in mice liver induced by HFD.

Metabolites	CK vs. NK		NK vs. FH		CK vs. FH		CK vs. YK		NK vs. YK	
	Log_2_FC	FC	Log_2_FC	FC	Log_2_FC	FC	Log_2_FC	FC	Log_2_FC	FC
Taurodeoxycholic acid sodium salt	1.43↑	2.69			−1.90↓	0.27				
Tauroursodeoxycholic Acid			−1.88↓	0.27						
Taurochenodesoxycholic Acid	1.00↑	2.01	−1.90↓	0.27						
Hyodeoxycholic Acid							−1.07↓	0.48	−1.88↓	0.27
Glycoursodeoxycholic Acid			−1.39↓	0.38						
Glycocholic Acid	−1.22↓	0.43					−2.24↓	0.21	−1.02↓	0.49
Deoxycholic Acid			1.04↑	2.06			−1.60↓	0.33	−1.46↓	0.36
Chenodeoxycholic Acid			1.06↑	2.09			−1.54↓	0.34	−1.43↓	0.37
Taurodeoxycholic Acid sodium salt									−1.53↓	0.35
D-Sorbitol	1.04↑	2.06	−1.42↓	0.37			1.77↑	3.40		
D-Fructose	1.03↑	2.04	−1.27↓	0.41			1.37↑	2.59		
D-Galactose	1.18↑	2.26	−1.43↓	0.37			1.71↑	3.28		
D-Glucose	1.24↑	2.36	−1.53↓	0.35			1.79↑	3.46		
D-Mannose	1.31↑	2.48	−1.56↓	0.34			1.67↑	3.17		
D-Sucrose			−3.89↓	0.07	−1.30↓	0.41	4.67↑	25.43		
D-Trehalose	4.12↑	17.37	−3.78↓	0.07			5.08↑	33.73		
D-Glucose 6-Phosphate	2.73↑	6.65	−1.75↓	0.30			3.64↑	12.49		
L-Fucose							−1.35↓	0.39		
L-Rhamnose							−1.08↓	0.47		
Maltose							5.48↑	44.70		
D-Sedoheptuiose 7-Phosphate	1.00↑	2.00					1.30↑	2.47		
D-Glucopyranose			−1.59↓	0.33						
D-Fructose 6-Phosphate -Disodium Salt	2.76↑	6.77	−1.62↓	0.32	1.14↑	2.20	3.71↑	13.13		
D-Fructose−1,6-Biphos- phate-Trisodium Salt	2.15↑	4.45	−2.01↓	0.25			2.75↑	6.70		
N-Acetylglucosamine 1-Phosphate					1.06↑	2.08				
3′-Sialyllactose	3.14↑	8.84	−2.11↓	0.23	1.03↑	2.04	3.08↑	8.45		
Galactaric Acid					1.17↑	2.25				
Hydroxyphenyllactic Acid	1.47↑	2.78					1.13↑	2.19		
MARMESIN	−1.57↓	0.34	1.16↑	2.23						
L-Erythrulose	1.58↑	2.99	−1.68↓	0.31			2.19↑	4.55		
D-Mannose 6-phosphate	2.75↑	6.72	−1.67↓	0.31	1.07↑	2.11	3.73↑	13.31		
D-Fructose 6-phosphate	2.71↑	6.56	−1.72↓	0.30			3.59↑	12.06		
Formononetin	−12.99↓	0.00	11.32↑	2561.81	−1.67↓	0.31	−12.99↓	0.00		
D-Sucrose	3.56↑	11.80							1.11↑	2.16
Lactobionic Acid					2.24↑	4.74	2.32↑	4.99		
Dulcitol							2.64↑	6.24	1.01↑	2.02
Lactose					−1.37↓	0.39	3.35↑	10.20		
Maltotriose	12.40↑	5418.78	−5.19↓	0.03	7.21↑	147.99	14.06↑	17110.83	1.66↑	3.16
N-Acetyl-D-Glucosamine					1.12↑	2.18				
Raffinose	12.57↑	6062.72	−5.88↓	0.02	6.69↑	103.22	14.18↑	18620.56	1.62↑	3.07
D-Mannitol			−1.83↓	0.28			2.41↑	5.33		
D(-)-Threose					1.31↑	2.48				
Octadecatrienoic Acid	−1.41↓	0.38								
Thromboxane B2							−2.36↓	0.19	−2.58↓	0.17
Prostaglandin D2							−3.44↓	0.09	−3.20↓	0.11
Eicosapentaenoic Acid	−3.27↓	0.10	1.30↑	2.46	−1.98↓	0.25	−4.98↓	0.03	−1.71↓	0.31
Eicosatetraenoic Acid	−4.52↓	0.04	2.83↑	7.09	−1.69↓	0.31	−6.83↓	0.01	−2.32↓	0.20
Eicosatrienoic Acid							−1.92↓	0.26	−1.71↓	0.31
Docosahexaenoic Acid	−4.79↓	0.04	2.71↑	6.54	−2.08↑	0.24	−6.82↓	0.01	−2.04↓	0.24
12-Hydroxyeicosane tetraenoic acid	−2.29↓	0.20	1.48↑	2.79			−3.09↓	0.12		
Lysopc 16:1					−1.09↓	0.47				
Lysopg 18:1	1.34↑	2.53								
Lysope 18:1	1.29↑	2.44								
Lysopc 20:2							1.15↑	2.21		
Lysopa 18:0	1.79↑	3.47			2.15↑	4.42	2.03↑	4.08		
Cis-11,14-Eicosadienoic Acid (C20:2)	1.20↑	2.30			1.28↑	2.44	1.15	2.21		
Arachidic Acid(C20:0)	−1.37↓	0.39					−1.76↓	0.30		
3-Methylglutaric Acid							−1.38↓	0.39		
Tetradecanedioic Acid	−1.30↓	0.41					−1.96↓	0.26		
Hexadecanedioic Acid							−1.78↓	0.29		
PE(18:1(9Z)/0:0)	1.37↑	2.58					1.40↑	2.65		
Punicic Acid	−2.21↓	0.22	1.26↑	2.39			−2.61↓	0.16		
Stearidonic Acid	−1.56↓	0.34			−1.67↓	0.31			1.71↑	1.71
p-Mentha-1,3,8-triene	−1.05↓	0.48							1.63↑	1.63
2,2-Dimethyl Succinic Acid							−1.31↓	0.40		
2-Methylbutyroylcarnitine			−1.56↓	0.34	−1.63↓	0.32				

The significant regulation of potential metabolites in the liver of mice induced by HFD shown in Table 2. Metabolites with “↑/↓” means increased or decreased. FC means “Fold Change”.

## Data Availability

The data that support the findings of this study are available from the corresponding author upon reasonable request.
